# Outcomes of Blunt Suprahepatic Vena Cava Injuries: A Retrospective Study from a Single Trauma Center in Korea

**DOI:** 10.3390/jcm15103652

**Published:** 2026-05-09

**Authors:** Donghwan Choi, Sang-Hyun Lim, Jonghwan Moon

**Affiliations:** 1 Department of Trauma and Acute Care, Ajou University School of Medicine, Suwon 16499, Republic of Korea; claptonc@naver.com; 2 Department of Thoracic and Cardiovascular Surgery, Ajou University School of Medicine, Suwon 16499, Republic of Korea; goodsurgeon@hanmail.net

**Keywords:** suprahepatic inferior vena cava, cardiopulmonary bypass, blunt trauma, abdominal vascular injury

## Abstract

**Background/Objectives:** Blunt suprahepatic inferior vena cava (SHIVC) injury is a rare and highly lethal condition associated with severe thoracoabdominal trauma. This study describes the clinical characteristics and outcomes of SHIVC injuries treated at a single institution. **Methods:** We retrospectively reviewed patients with blunt SHIVC injury treated between January 2014 and September 2023. Demographics, injury characteristics, management strategies, and outcomes were analyzed descriptively. The primary outcome was in-hospital mortality. Given the small sample size, statistical analyses were exploratory. **Results:** Ten patients were identified (mean age: 47 ± 17 years; mortality: 40%; ISS: 43 ± 19). On admission, 50% presented with systolic blood pressure < 90 mmHg and a mean Glasgow Coma Scale score of 7 ± 5. Non-survivors had lower systolic blood pressure (70 ± 12 vs. 115 ± 34 mmHg, *p* = 0.05), lower GCS scores (3 ± 0 vs. 9 ± 5, *p* = 0.02), and worse base excess (−20.1 ± 5.8 vs. −7.8 ± 7.1) than survivors. Surgical intervention was performed in 9 patients, while 1 was managed nonoperatively. Common associated injuries included right atrial injury (70%), liver injury (50%), and diaphragm injury (30%). Four patients received intraoperative circulatory support; two treated with cardiopulmonary bypass survived, whereas those treated with extracorporeal membrane oxygenation died. No definitive conclusions can be drawn regarding treatment effectiveness due to the limited sample size. **Conclusions:** Outcomes appear strongly influenced by initial physiological status and injury severity. This study is descriptive and hypothesis-generating; further multicenter studies remain warranted to define optimal management strategies.

## 1. Introduction

Inferior vena cava (IVC) injuries are associated with high fatality rates, and suprahepatic inferior vena cava (SHIVC) injuries have an exceptionally high fatality rate [1,2,3,4,5,6,7]. SHIVC injuries present substantial challenges that require a multidisciplinary approach, including simultaneous thoracic and abdominal surgery, as well as extensive blood transfusion [8]. This complexity is largely attributed to the anatomical location of the SHIVC, in which over two-thirds of systemic venous return passes through the vessel; therefore, injury at this level rapidly leads to exsanguination and severely limits surgical exposure [4,9,10]. In addition, a key unresolved clinical dilemma in the management of SHIVC injury is the selection of optimal circulatory support and operative strategy. In particular, the indications and timing for cardiopulmonary bypass (CPB), extracorporeal membrane oxygenation (ECMO), and alternative adjuncts such as atriocaval shunting remain poorly defined and are largely based on institutional preference, resource availability, and intraoperative judgment rather than standardized guidelines [3,4,6,10,11,12,13]. Consequently, the decision-making process varies significantly across centers and clinical scenarios, particularly in hemodynamically unstable patients requiring emergent intervention versus those in whom definitive repair under controlled conditions is feasible.

Despite multiple reported treatment strategies, the optimal surgical and resuscitative approach for SHIVC injury remains unclear. We hypothesized that using CPB in selected cases is associated with improved survival, compared with other treatment modalities; however, this remains controversial due to the rarity and heterogeneity of these injuries. Therefore, this study aimed to analyze the clinical characteristics, treatment decision-making process, and outcomes of SHIVC injuries managed at our institution and contextualize our findings within the existing literature.

## 2. Materials and Methods

### 2.1. Data Collection

This retrospective study was conducted at a single trauma center in Korea. Patients with radiologically or surgically confirmed traumatic IVC injuries were identified from the institutional trauma database between January 2014 and September 2023. In this study, SHIVC injury was defined as any traumatic injury involving the IVC segment above the hepatic veins and extending to the intrapericardial IVC. This definition was based on a combination of operative findings and contrast-enhanced computed tomography (CT) interpretation, in accordance with previously described anatomical classifications of caval injury [14,15]. Data collected included baseline demographic information, injury mechanisms, Glasgow coma scale (GCS) score, first 24-h transfusion volumes, injury severity score (ISS), arterial blood gas results at arrival, and international normalized ratio (INR). Medical records were reviewed to document resuscitation methods performed in the emergency department. Operative records were analyzed to extract details of surgical techniques, estimated blood loss, and surgery time for SHIVC injuries and associated injuries.

### 2.2. Treatment Decision-Making and Study Outcomes

Treatment decisions were made by a multidisciplinary trauma team that included trauma and cardiothoracic surgeons, based on hemodynamic status, injury location, and intraoperative findings. Patients with suspected intrapericardial IVC injury or cardiac involvement—particularly in the presence of hemopericardium—underwent early cardiothoracic consultation.

The use of cardiopulmonary bypass (CPB) or extracorporeal membrane oxygenation (ECMO) was determined intraoperatively. CPB was preferred when definitive repair under direct visualization was required and when resources and personnel were readily available. ECMO was considered in cases that require rapid initiation of circulatory support or when CPB was not immediately feasible. Importantly, the decision between CPB and ECMO was largely influenced by the patient’s initial hemodynamic status and overall injury severity, with more unstable patients or those with profound physiological derangement being likely to undergo emergent support strategies. This introduces an inherent risk of selection bias when comparing outcomes between groups.

The primary outcome of this study was in-hospital mortality. Secondary outcomes included transfusion requirements within 24 h, operative variables (door to surgery time and operative duration), and postoperative complications.

### 2.3. Statistical Analysis

Normality was assessed using the Kolmogorov–Smirnov test. Given the small sample size, statistical analyses were primarily descriptive. Continuous variables are presented as mean ± standard deviation or median (interquartile range), while categorical variables are given as counts and percentages. Any inferential testing was considered exploratory and hypothesis-generating. All analyses were conducted using the R package (moonBook 0.3.1). The significance threshold was set at *p* < 0.05.

### 2.4. Ethics Statement

This study was conducted in accordance with the Declaration of Helsinki, and approved by the Institutional Review Board of our institution. Informed consent was waived due to the retrospective design of the study.

## 3. Results

### 3.1. Demographics

Between March 2014 and September 2023, 10 patients with SHIVC injuries were identified in the institutional trauma database. The mean patient age was 47 years, with eight male and two female patients. All injuries resulted from blunt trauma. Among these patients, seven (70%) presented directly to the hospital, whereas 3 (30%) were transferred from other facilities (Table 1). The mean ISS was 43, with survivors and non-survivors having a mean ISS of 35 and 57, respectively. The mean time from the accident to arrival at the emergency department was 69 min for survivors and 94 min for non-survivors.

### 3.2. On-Arrival Variables and Laboratory Results

The mean systolic blood pressure (SBP) of survivors was 115 ± 34 mmHg, with a shock rate of 17% (*n* = 1). Conversely, all non-survivors had an SBP < 90 mmHg (*p* = 0.05) and their GCS score was lower than that of survivors (3 ± 0 vs. 9 ± 5, *p* = 0.02). Laboratory results showed that lactate levels (14.2 ± 7.2 vs. 6.7 ± 5.0 mmol, *p* = 0.08), base excess (−20.1 ± 5.8 vs. −7.8 ± 7.1 mmol, *p* = 0.02), and INR (3.1 ± 2.6 vs. 1.2 ± 0.2, *p* = 0.49) were worse in non-survivors than in survivors (Table 1 and Table 2).

### 3.3. Treatment for SHIVC Injury and Associated Injuries

**Figure 1:**
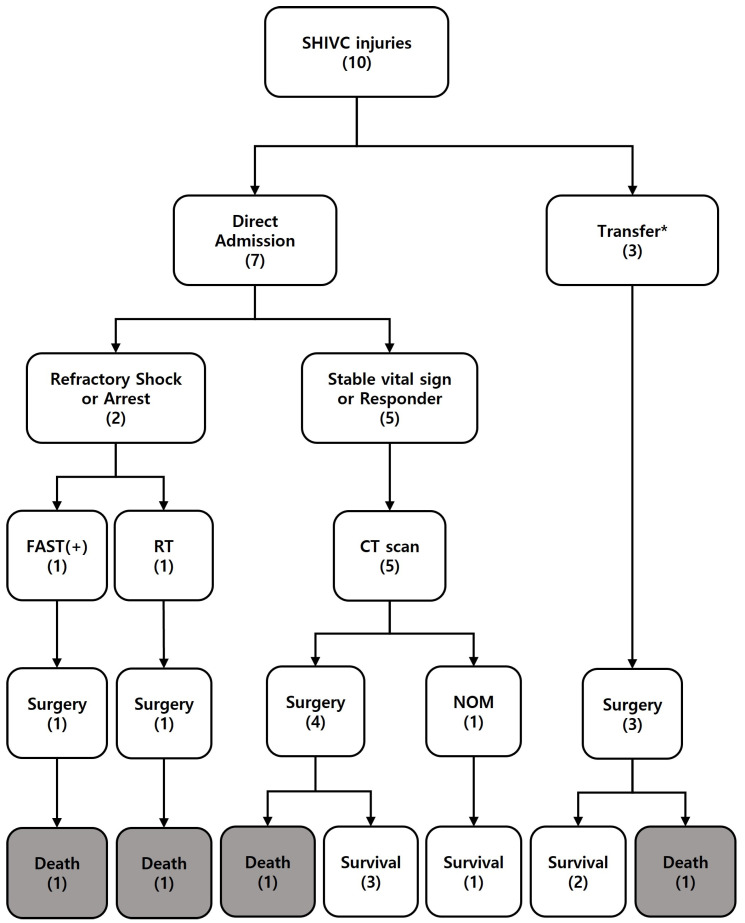
Hospital course of SHIVC injury. * All patients were transferred after the CT scan. SHIVC, suprahepatic inferior vena cava; CT, computed tomography; FAST, focus assessment of sonography for trauma; RT, resuscitative thoracotomy; NOM, non-operative management.

**Figure 2:**
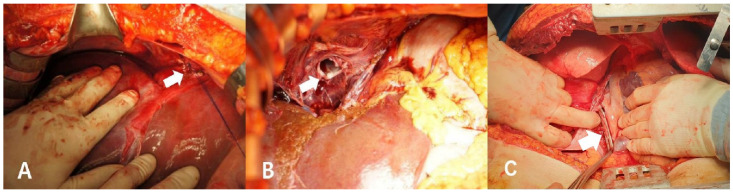
Surgical findings of SHIVC injuries. (**A**) SHIVC injury after primary repair in Patient No. 1. (**B**) Transected SHIVC in Patient No. 7. (**C**) Longitudinal tearing of intrapericardial segment of SHIVC with Satinsky clamps in Patient No. 10. SHIVC, suprahepatic inferior vena cava. The white arrow indicates the injured inferior vena cava.

Surgical intervention was performed on nine patients, whereas one patient underwent non-operative management (NOM) (Figure 1 and Figure 2). The time from hospital arrival to the operating room was longer for survivors than for non-survivors (220 ± 49 vs. 38 ± 18 min, *p* < 0.01). Surgery time was also longer for survivors than for non-survivors (155 [80–195] min vs. 56 [45–59] min, *p* = 0.02). There was no difference in estimated blood loss between survivors and non-survivors (4040 ± 2027 vs. 4200 ± 2072 mL, *p* = 0.91) (Table 1 and Table 3). The most common associated injury was right atrial injury (*n* = 7, 70%), followed by liver injury (*n* = 5, 50%) and diaphragm injury (*n* = 3, 30%) (Table 3 and Table 4). Primary repair for IVC injury was performed in eight patients, whereas one patient underwent patch repair. Among the four patients treated with cardiopulmonary support, two received CPB and two ECMO. Survival was observed in those who received CPB, whereas the patients treated with ECMO died. Given the small sample size, these findings should be interpreted as descriptive observations rather than indicative of comparative effectiveness (Table 3).

### 3.4. Transfusion and Outcomes

Of the 10 patients with SHIVC injuries, nine underwent blood transfusions within the first 24 h, excluding one patient who received NOM. The 24 h packed red blood cell and fresh frozen plasma transfusion volumes were higher in survivors than in non-survivors. Platelet transfusions in survivors averaged 8 ± 6 units, showing a difference (Table 1 and Table 4). These findings, which present descriptive comparisons between survivors and non-survivors, should not be interpreted as indicating a causal relationship between transfusion volume and survival outcomes.

Two survivors developed complications, including pneumonia and pulmonary thromboembolism. Moreover, one patient was diagnosed with IVC stricture 2 years post-surgery. The mean length of stay for survivors was 35 days, with a mean intensive care unit stay of 14 days (Table 5).

## 4. Discussion

Blunt IVC injury is a highly fatal condition, with reported mortality rates reaching 70–80% [7,14]. The higher mortality observed in non-survivors is likely attributed to the profound physiological collapse at presentation rather than differences in subsequent management [7,16]. Studies indicate that the closer the level of injury is to the heart, the higher the mortality rate, making SHIVC injuries the most lethal subtype of IVC trauma [2,7,17]. In the present study, 10 patients were diagnosed with SHIVC injury, with an overall mortality rate of 40%. This observed rate is lower than previously reported mortality rates in the literature. For example, Netto et al. reported that all patients with blunt SHIVC injuries in their study died [16]. Huerta et al., in their analysis of 818 IVC injuries, found a mortality rate of 78% for SHIVC injuries [2].

Experienced trauma surgeons have noted that due to massive bleeding associated with suprahepatic and retrohepatic vena cava injuries, direct surgical access to the damaged area is difficult, requiring multidisciplinary treatment to maintain hemodynamic stability during surgery [5,8,13]. Advances in treatment techniques have led to successful outcomes in many cases [5,6,18,19,20]. Previously reported cases of blunt suprahepatic inferior vena cava injuries are summarized in Supplementary Table S1. In this small cohort, CPB was used in patients who survived, whereas ECMO was used in those who did not; however, given the very limited sample size in this study, no definitive conclusions can be drawn regarding the comparative effectiveness of these approaches [4,5,6,13,18,19,20,21,22]. Consistent with the findings of Oh et al., two patients in our study who received CPB survived, whereas two patients treated with ECMO died [6]. When using an ECMO system with a closed circuit, opening the pericardium may lead to rapid blood loss from the ruptured IVC, allowing air to enter through the drainage catheter and subsequently compromise the ECMO system. Conversely, CPB maintains perfusion through a reservoir even during significant blood loss, despite the necessity for systemic heparinization. This difference likely contributed to the higher survival rate observed with CPB. These findings suggest that survival in SHIVC injury is primarily determined by the degree of initial hemorrhagic shock and rapidity of venous return compromise rather than the specific circulatory support. In our limited cohort, CPB was associated with survival, whereas ECMO was not; however, this observation should be interpreted with caution due to potential selection bias and the small sample size.

Blunt SHIVC injuries commonly involve the intrapericardial segment, including the heart, often resulting in hemopericardium [5,8,13,16]. Oh et al. reported that SHIVC injuries were primarily intrapericardial segments, with cardiac tamponade diagnosed in all cases due to hemopericardium [6]. Similarly, Netto et al. reported that pericardial fluid was confirmed through focused assessment with sonography in trauma, alongside injuries to the IVC and right atrium in the intrapericardial segment [16]. In the present study, right atrium and intrapericardial segment injuries were observed in 70% of SHIVC injuries. Therefore, it is essential to recognize that signs suggestive of SHIVC injuries, such as pericardial fluid and hemopericardium, during resuscitation warrant a multidisciplinary surgical approach, including cardiothoracic specialist involvement.

Another key feature of blunt SHIVC injuries is the high ISS, frequently associated with organ injuries such as those to the diaphragm, liver, and spleen [6,13]. Tsai et al. and Paulo et al. also reported that SHIVC injuries frequently involve liver and diaphragm injuries, with an ISS of 27 and 54 points, respectively [13,14]. Furthermore, Oh et al. noted liver, spleen, diaphragm, and pelvis injuries in SHIVC cases, with a mean ISS of 32 points [6]. In the present study, similar injuries were observed, including liver injury (50%) and diaphragm injury (30%), as well as other associated injuries involving the kidney, aorta, femur, and brain. The mean ISS was 47 points, with all associated injuries requiring surgical or interventional treatment. Although the ISS for non-survivors was higher than those for survivors (57 ± 21 vs. 34 ± 12), statistical significance of this difference could not be confirmed due to the small sample size of the study.

Predictors of mortality in IVC injuries include GCS score, injury mechanism, hypotension, ISS, injury location, and transfusion volume [1,17,23,24,25,26]. Although the small sample size of this study limited the identification of independent risk factors, differences were observed in the SBP, GCS score, and base excess upon admission. Platelet transfusion volume, time to the operating room, and operative duration showed statistical significance; however, these results should be interpreted cautiously, as they are likely influenced by survivorship and early mortality bias. Patients who die early may not survive long enough for prolonged resuscitation, extensive diagnostic evaluation, or extended operative intervention. Consequently, the shorter time to surgery and operative duration observed in non-survivors more plausibly reflect the severity of initial physiological compromise and rapid clinical deterioration, rather than any benefit associated with expedited or abbreviated management. These variables should therefore be interpreted as surrogate markers of injury severity and physiological derangement at presentation, rather than indicators of treatment effect. Consistent with the results of prior research, our findings further support an association between initial physiological status and patient outcomes.

This study has several important limitations. First, the very small sample size (*n* = 10) substantially limits statistical power and precludes meaningful comparative analysis. Thus, the observed differences between survivors and non-survivors may reflect random variation rather than true associations. Second, the retrospective, single-center design introduces inherent limitations, including incomplete control over confounding variables and limited generalizability to other trauma systems or patient populations. Third, there was considerable clinical heterogeneity in injury severity, physiological status on presentation, and associated injuries, limiting the direct comparison between treatment strategies, as management decisions were highly individualized and context-dependent. Fourth, selection bias is another likely limitation, as treatment strategies—CPB or ECMO—were determined based on patient condition and surgeon judgment. Consequently, differences in outcomes may reflect underlying patient selection rather than treatment effects. Fifth, survivorship bias must be considered. Patients who died early may not have survived long enough for prolonged resuscitation or complex operative interventions. Therefore, longer time to surgery or higher transfusion volumes observed in survivors likely reflect survival duration rather than a beneficial effect of these factors. Taken together, these limitations restrict causal inference, and the findings should be interpreted as descriptive and hypothesis-generating rather than indicative of treatment effectiveness or superiority.

Nevertheless, this study has several notable strengths. First, it focuses on an exceptionally rare and highly lethal injury pattern, providing one of the few contemporary institutional experiences with SHIVC injuries. Second, it details operative strategies, perioperative management, and multidisciplinary approaches, potentially informing clinical decision-making in similar emergent settings. Third, it reflects the evolving management of these injuries within a modern trauma system. Further large-scale, multicenter studies remain warranted to validate these findings and better define optimal management strategies for SHIVC injuries.

In conclusion, SHIVC injuries are associated with multiple concomitant organ injuries and require a multidisciplinary management approach. Advances in trauma care, resuscitation strategies, and diagnostic modalities may contribute to improved outcomes in these critically injured patients.

In this small and heterogeneous cohort, differences in management strategies, including CPB, were observed among survivors and non-survivors. However, given the limited sample size and potential selection bias, the findings of this study should be interpreted as hypothesis-generating rather than indicative of causal or comparative effectiveness. Future large-scale, multicenter studies are warranted to further investigate optimal management strategies for SHIVC injuries.

## Figures and Tables

**Table 1:** Demographics and baseline information.

	Survival (*n* = 6)	Non-Survival (*n* = 4)	Total (*n* = 10)	*p*
Age (year)	44 ± 16	52 ± 20	47 ± 17	0.54
Sex (M/F)	4/2	4/0	8/2	0.63
Admission (Direct/Transfer)	4/2	3/1	7/3	1.00
ISS	34 ± 12	57 ± 21	43 ± 19	0.06
Length of ED stay (min)	40 [35–77]	64 [40–148]	42 [36–85]	0.35
On admission variables				
Systolic blood pressure (mmHg) *	115 ± 34	70 ± 12	97 ± 35	0.04 *
<90 mmHg *	1 (17%)	4 (100%)	5 (50%)	0.05 *
Pulse rate (min)	100 ± 18	92 ± 19	97 ± 18	0.55
Glasgow coma scale *	9 ± 5	3 ± 0	7 ± 5	0.02 *
Laboratory findings				
Lactate (mmol)	6.7 ± 5.0	14.2±7.2	9.7 ± 6.8	0.08
Base excess (mmol) *	−7.8 ± 7.1	−20.1±5.8	−12.7 ± 9.0	0.02 *
INR	1.2 ± 0.2	3.1±2.6	1.7 ± 1.3	0.49
First 24-h transfusion (unit)				
PRBC	14 ± 10	10 ± 1	13 ± 8	0.39
FFP	15 ± 12	10 ± 3	13 ± 10	0.31
Platelet *	8 ± 6	0 ± 0	4 ± 6	0.03 *
Surgery information (*n* = 9)				
Door to operating room (min) *	220 ± 49	38 ± 18	140 ± 103	<0.01 *
Surgery time (min) *	155 [80–195]	56 [45–59]	75 [58–155]	0.02 *
EBL for surgery (mL)	4040 ± 2027	4200 ± 2072	4111 ± 1916	0.91

F, female; M, male; ISS, injury severity score; ED, emergency department; INR, international normalized ratio; PRBC, packed red blood cells; FFP, fresh frozen plasma; EBL, estimated blood loss. * *p* < 0.05.

**Table 2:** Baseline information of patients with SHIVC injuries.

No	Age (Year)	Sex	Injury Mechanism	Admission	ISS	CT	FAST	Time to ED (min)	SBP (mmHg)	Pulse Rate (Beats/min)	GCS	Lactate (mmol)	BE (mmol)	INR
1	29	F	MVA	Transfer	36	O		197	92	93	4	6.0	−3.5	1.63
2	32	M	MVA	Transfer	25	O	O (+)	77	75	100	3	15.1	−19.0	1.06
3	51	F	MVA	Direct	41	O	O (+)	35	130	110	13	9.8	−13.6	0.99
4	30	M	MCA	Direct	17	O	O (−)	26	158	86	14	2.2	−0.1	1.27
5	60	M	Fall	Direct	50	O	O (−)	41	144	80	9	2.3	−6.3	1.32
6	64	M	MVA	Direct	38	O	O (−)	38	90	130	12	5.0	−4.0	1.12
7	48	M	MVA	Direct	75	O	O (+)	85	79	73	3	24.0	−26.1	- *
8	58	M	MVA	Transfer	42	O		211	72	80	3	10.3	−18.6	- *
9	75	M	MVA	Direct	75		O (+)	43	76	109	3	7.7	−12.8	1.29
10	26	M	Fall	Direct	35		O (−)	36	52	108	3	14.8	−23.0	4.93

ISS, injury severity score; CT, computed tomography; FAST, focus assessment with sonography in trauma; ED, emergency department; SBP, systolic blood pressure; GCS, Glasgow coma scale; BE, base excess; INR, international normalized ratio; F, female; M, male; MVA, motor vehicle accident; MCA, motorcycle accident. * Reported as a panic result because the value exceeded the measurable range.

**Table 3:** Information of surgery and procedures.

No	Time to Surgery (min)	Surgery Time (min)	EBL for Surgery (mL)	Treatment Methods for IVC Injury	Associate Injuries (AIS ≥ 3)	Procedures for Combined Injury
1	208	195	6000	primary repair	Diaphragm, Liver	Diaphragm repair, Liver suture, Segmentectomy of liver, Perihepatic packing, Balloon angioplasty
2	233	440	1000	patch repair, CPB	RA, Tricuspid valve	RA repair, Tricuspid valvuloplasty & chordal repair, IVC filter insertion
3	190	155	5000	primary repair, CPB	RA, Diaphragm, Liver	RA repair, Diaphragm repair, Liver suture, Perihepatic packing
4	-	-	-	NOM	Liver, Kidney	Angioembolization
5	299	80	3000	primary repair	RA, Aorta, Diaphragm, mesentery, SAH	RA repair, Mesentery suture, TEVAR
6	172	75	5200	primary repair	RA, femur fractures	RA repair, ORIF
7	62	35	4000	primary repair	Liver	Liver suture, Perihepatic packing
8	24	55	3800	primary repair, ECMO	RA, LA, Liver	RA repair, Liver suture, Perihepatic packing
9	25	60	7000	primary repair, ECMO	RA	RA repair
10	43	58	2000	primary repair	RA, Extensive lung laceration	RA repair, Lung repair, Resuscitative thoracotomy, and trans-aortic clamp

EBL, estimated blood loss; IVC, inferior vena cava; AIS; abbreviated injury score; CPB, cardiopulmonary bypass; RA, right atrium; NOM, non-operative management; SAH, subarachnoid hemorrhage; TEVAR, Thoracic Endovascular Aneurysm Repair; ORIF, open reduction and internal fixation; ECMO, extracorporeal membrane oxygenation.

**Table 4:** Associate injuries.

Associate Injuries (AIS ≥ 3)	*n* = 10
Thorax	
Right atrium	7
Diaphragm	3
Lung	1
Aorta	1
Left atrium	1
Abdomen	
Liver	5
Kidney	1
Mesentery	1
Brain	1
Femur	1

AIS, abbreviated injury score.

**Table 5:** Clinical results of SHIVC injuries.

No	24-h PRBC	24-h FFP	24-h PLT	Complications	Hospital LOS	ICU LOS	Survival/Death
1	19	16	8	IVC stricture (2 years after)	44	9	Survival
2	7	6	16	Pneumonia, PTE, DVT	46	28	Survival
3	24	28	12	Cerebral infarction	26	12	Survival
4	0	0	0	-	12	2	Survival
5	25	30	8	PTE	37	17	Survival
6	11	11	1	Pneumonia	43	16	Survival
7	12	8	0	-	1	1	Death
8	10	12	0	-	1	1	Death
9	10	12	0	-	1	1	Death
10	10	6	0	-	1	1	Death

PRBC, packed red blood cells; FFP, fresh frozen plasma; PLT, platelet; LOS, length of stay; ICU, intensive care unit; IVC, inferior vena cava; PTE, pulmonary thromboembolism; DVT, deep vein thrombosis.

## Data Availability

The raw data supporting the conclusions of this article will be made available by the authors on request.

## Supplementary Materials

The following supporting information can be downloaded at: https://www.mdpi.com/article/10.3390/jcm15103652/s1, Table S1: Literature review of blunt supra-hepatic inferior vena cava injuries [2,4,5,6,13,14,16,18,19,20,21,22,27].

## Abbreviations

The following abbreviations are used in this manuscript:

IVC	Inferior vena cava
SHIVC	Suprahepatic inferior vena cava
CPB	Cardiopulmonary bypass
ECMO	Extracorporeal membrane oxygenation
GCS	Glasgow coma score
ISS	Injury severity score
INR	International normalized ratio
SBP	Systolic blood pressure
NOM	Non-operative management
PRBC	Packed red blood cell
FFP	Fresh frozen plasma
CT	Computed tomography
FAST	Focus assessment with sonography in trauma
ED	Emergency department
BE	Base excess
MVA	Motor vehicle accident
MCA	Motorcycle accident
EBL	Estimated blood loss
AIS	Abbreviated injury score
RA	Right atrium
PLT	Platelet
LOS	Length of stay
ICU	Intensive care unit
PTE	Pulmonary thromboembolism
DVT	Deep vein thrombosis
