# Supplementary material for: Outcomes of Blunt Suprahepatic Vena Cava Injuries: A Retrospective Study from a Single Trauma Center in Korea
Source: J Clin Med. 2026 May 9;15(10):3652. doi: 10.3390/jcm15103652 (PMC13207943; doi:10.3390/jcm15103652)
Supplement: Supplementary file 1 [file jcm-15-03652-s001.zip › jcm-4261947-supplementary.pdf]

Supplementary Table S1. Literature review of blunt supra-hepatic inferior vena cava injuries

Author	Year (publish)	Country	Type of injury	Age	ISS	n	Death	Procedure
LAUNOIS [18]	1989-case report	USA	blunt, TA	29	-	1	0	CPB
Netto [16]	2006-case series	Canada	blunt, TA	46	-	2	2	-
Huerta [2]	2006-original (IVC injury, n=36)	USA	blunt and penetrating	-	-	5	5	-
Prabhu [19]	2007-case report	India	blunt, TA	50	-	1	1	CPB
Marino [20]	2008-case report	USA	blunt, TA	17	-	1	0	CPB
Kaemmerer [21]	2011-case report	Germany	blunt, TA	15	-	1	0	CPB
Tsai [14]	2016-case series	USA	blunt	34	27	0	0	-
Salloum [22]	2016-case report	France	blunt, TA	33	-	0	0	CPB
Kim [4]	2018-case report	Korea	blunt, TA	43	-	0	0	CPB
Martins [13]	2021-case report	USA	blunt, TA	57	54	0	0	CPB
Rooke [5]	2021-case report	USA	blunt, TA	32	-	0	0	CPB
Oh [6]	2023-case series	Korea	blunt, TA	58	32	1	1	ECMO-die CPB-survive
Park [27]	2023-original (IVC injury, n=16)	Korea	blunt and penetrating	-	34	1	1	-
Choi	present study	Korea	blunt	47	43	10	4	ECMO-die CPB-survive

ISS, injury severity score; TA, traffic accident; CPB, cardiopulmonary bypass; IVC, inferior vena cava; ECMO, extracorporeal membrane oxygenation
